# Pathogenesis of Important Virulence Factors of *Porphyromonas gingivalis* via Toll-Like Receptors

**DOI:** 10.3389/fcimb.2019.00262

**Published:** 2019-07-18

**Authors:** Lu Jia, Nannan Han, Juan Du, Lijia Guo, Zhenhua Luo, Yi Liu

**Affiliations:** ^1^Laboratory of Tissue Regeneration and Immunology and Department of Periodontics, Beijing Key Laboratory of Tooth Regeneration and Function Reconstruction, School of Stomatology, Capital Medical University, Beijing, China; ^2^Department of Orthodontics, School of Stomatology, Capital Medical University, Beijing, China

**Keywords:** *Porphyromonas gingivalis*, Toll-like receptors, lipopolysaccharide, gingipains, fimbriae, virulence factor, periodontitis

## Abstract

Periodontitis is a common intraoral infection and is inextricably linked to systemic diseases. Recently, the regulation between host immunologic response and periodontal pathogens has become a hotspot to explain the mechanism of periodontitis and related systemic diseases. Since *Porphyromonas gingivalis* (*P. gingivalis*) was proved as critical periodontal pathogen above all, researches focusing on the mechanism of its virulence factors have received extensive attention. Studies have shown that in the development of periodontitis, in addition to the direct release of virulent factors by periodontal pathogens to destroy periodontal tissues, over-low or over-high intrinsic immune and inflammatory response mediated by Toll-like receptors (TLRs) can lead to more lasting destruction of periodontal tissues. It is very necessary to sort out how various cytopathic factors of *P. gingivalis* mediate inflammation and immune responses between the host through TLRs so as to help precisely prevent, diagnose, and treat periodontitis in clinic. This review summarizes the role of three most widely studied pathogenic factors produced by *P. gingivalis* (lipopolysaccharide, gingipains, pili) and their interactions with TLRs at the cellular and molecular level in the progress of periodontitis.

## Introduction

Periodontitis refers to inflammatory pathological damage of the gums and periodontal support tissues, including the gums, alveolar bone, periodontal ligament, and cementum. Untreated periodontitis can cause the formation of deep periodontal pockets, which eventually lead to loosening of the teeth (Pihlstrom et al., 2005; Bostanci and Belibasakis, 2012). According to a survey by the World Health Organization, 10–15% of adults worldwide suffer from periodontitis (Petersen and Ogawa, 2012). The factor initiating periodontitis is biofilm plaques, in which *Porphyromonas gingivalis (P. gingivalis)*, a Gram-negative anaerobe and component of the “red complex” (categorized together with *Tannerella forsythia* and *Treponema denticola*, highly relevant to periodontitis), has been irrefutably shown to be the key pathogen underlying the pathogenesis of chronic periodontitis (CP) (Parahitiyawa et al., 2010). Furthermore, host inflammatory and immune responses to microbial communities change the subgingival environment, causing low-abundant key opportunistic pathogens such as *P. gingivalis* to become the dominant bacteria in the biofilm, thus breaking the homeostasis between symbiotic microorganisms and the host, promoting the development of periodontitis, and even triggering systemic diseases (Abdi et al., 2017). For example, animal experiments have been used to demonstrate that *P. gingivalis* can also colonize some distant organs, such as the coronary artery, placenta, liver, and even the brain, causing specific infections associated with the activation of Toll-like receptors (TLRs) (Olsen and Yilmaz, 2016; Huck et al., 2018). Based on these data, an increasing number of scholars are investing in molecular biology to explore the pathogenic mechanisms of *P. gingivalis* (Olsen et al., 2018). However, the specific pathogenic effect of *P. gingivalis* virulence factors remain incompletely understood, and many topics remain controversial.

The so-called virulence factors are molecules that cause damage to the host at different stages of the organism's (bacteria, viruses, fungi, and protozoa) life cycle, increasing their effectiveness. They mainly involve the following functions: (1) colonization in the host; (2) immune escape; (3) immunosuppression; (4) cellular entry and exit; (5) extraction of nutrients from the host; and (6) release of virulence factors (How et al., 2016). *Porphyromonas gingivalis* can produce various virulence factors, such as lipopolysaccharide (LPS), gingipains, fimbriae/pili, collagenase, (erythrocyte) lectins, capsule, protease, and superoxide dismutase, to evade the host immune defense system and destroy host periodontal tissues. Recent studies have confirmed that LPS, gingipains, and fimbriae/pili are the most important pathogenic substances of *P. gingivalis* and the most widely studied in the field of periodontitis, and each of these factors play key roles in the progression of periodontitis (Mysak et al., 2014).

Although periodontal pathogens play very important roles in the initial stage of periodontitis, directly destructing host periodontal tissues by releasing toxic factors and metabolites, the progression of periodontitis is regulated by mainly the interaction between the host immune response and periodontal pathogens. Because the indirect damage caused by host congenital and adaptive immunological responses that are overactivated or blocked by periodontal pathogens is more traumatic and lasts longer, either low reactivity or hyperresponsiveness of the immune response results in persistent periodontal tissue damage (Meyle et al., 2017). As we know, an organism first relies on innate immune responses to resist pathogenic microbial invasion, which is also a prerequisite for adaptive immunity. Typical pathogenic molecules are identified by multiple cell surface receptors, which are called pattern recognition receptors (PRRs) and include TLRs, NOD-like receptors (NLRs), C-type lectin receptors (CLRs), and RIG-I-like receptors (RLRs). After numerous pathogen-associated molecular patterns (PAMPs) are distinguished by these receptors, the intracellular signal transmission pathways are initiated, thereby stimulating the expression of inducible costimulators and releasing inflammatory factors, chemotactic factors, and interferons (except those of the gamma type), among others (Akira et al., 2006). TLRs are the most characteristic PRRs that activate and are widely expressed in multiple cell types, including neutrophils, macrophages, keratinocytes, and fibroblasts (Akira and Takeda, 2004). The key point in the innate immune response during the pathogenesis of periodontitis lies in the recognition between pathogenic factors and PRRs. A new case-control study comparing the relationship between early periodontitis and single nucleotide polymorphisms (SNPs) of TLRs, NLRs, and RLRs showed that TLR polymorphisms are associated with the susceptibility of adolescents to periodontitis (Leite et al., 2018). Some scholars performing a meta-analysis of the susceptibility of TLR4 polymorphism to periodontitis revealed that TLR4 C>G (rs7873784) may be associated with CP in the Asian population and be transmitted to the next generation in a recessive form (Jin et al., 2016). However, controversy exists regarding this topic (Song et al., 2013; Chrzeszczyk et al., 2015).

This review focuses specifically on how three critical *P. gingivalis* pathogenic factors explicitly contribute to the pathogenesis of periodontitis and how they mediate innate immunoinflammatory host responses via different TLRs.

## Effect of LPS on *P. gingivalis* Virulence

### Heterogeneity of LPS

**Figure 1:**
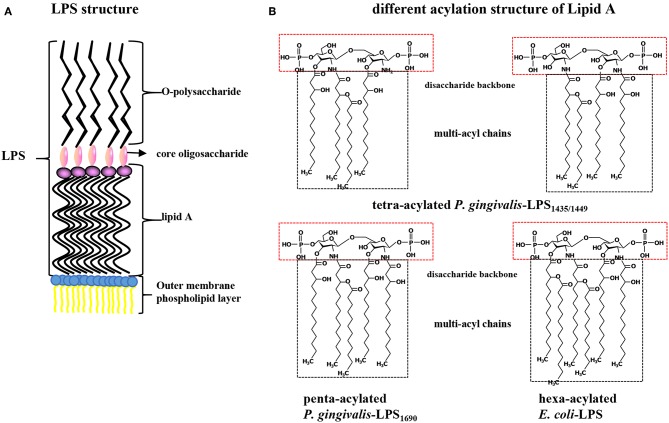
**(A)** Structure diagram of *P. gingivalis*-LPS. LPS is composed of lipid A (the conserved inner region without species specificity), core oligosaccharide (the bridge between lipid A and O-polysaccharide), and O-specific polysaccharide (a highly variable outer region) and located on bacterial outer membrane. **(B)** Contrast different acylation structure of *P. gingivalis* and *E. coli* LPS. Lipid A is composed of a phosphorylated β (1-6) D-glucosamine disaccharide backbone and multi-acyl chains acylated by fatty acids at specific positions. *P. gingivalis*-LPS with tetra-acylated chains is designated as LPS_1435/1449_ with its molecular weight of 1435 and 1449 Da, while the penta-acylated is named *P. gingivalis*-LPS_1690_ with a molecular weight of 1690 Da. The lipid A of *E. coli*-LPS is hexa-acylated.

*Porphyromonas gingivalis*-LPS, located on the lateral lobule of bacterial adventitia, is a bacterial endotoxin composed of lipid A (a conserved inner region without species specificity), core oligosaccharide (the bridge lipid A and O-polysaccharide), and O-specific polysaccharide (has a highly variable outer region) that has numerous biological activities and plays a strong pathogenic role in periodontal tissues (Schromm et al., 2000). Lipid A is the core factor underlying the immunological activity of LPS and is structurally composed of a phosphorylated β (1-6) D-glucosamine disaccharide backbone and multiacyl chains acylated by fatty acids at specific positions on the backbone (Dixon and Darveau, 2005; the schematic diagram of LPS structure shown in Figure 1A). *P. gingivalis*-LPS is released after the lysis of bacteria or as free vesicles outward from the outer membranes of living bacteria. These LPS-containing microvesicles, which function like “microbullets” that land on the host, further perpetuate the invasiveness of *P. gingivalis*, giving it the ability to destroy periodontal tissues and trigger inflammation (Zhang et al., 2015; Singhrao and Olsen, 2018). The basic chemical composition of *P. gingivalis*-LPS is nearly identical to that of a typical bacterial endotoxin. The key difference is that the LPS lipid A structure produced by *P. gingivalis* can undergo isomeric acylation by two modes (tetra-acylation and penta-acylation) depending on the environmental factors, such as the hemin levels, phosphate availability, and incubation temperatures, thus initiating differential immunoinflammatory responses (Rangarajan et al., 2017). Accordingly, *P. gingivalis*-LPS with tetra-acylated lipid A has been designated LPS_1435/1449_ based on its molecular weights of 1435 and 1449 Da, while the other molecule is named *P. gingivalis*-LPS_1690_ based on its molecular weight of 1690 Da (Curtis et al., 2011). Thus, TLR4 and TLR2 can be simultaneously activated after recognition of *P. gingivalis*-LPS, while *E. coli*-LPS can activate only TLR4 (Liu et al., 2008; Coats et al., 2009; different acylation structures of *P. gingivalis*-LPS and *E. coli*-LPS shown in Figure 1B). Therefore, the heterogeneous form of *P. gingivalis*-LPS is identified as a PAMP, and its regulatory role associated with host cell-specific TLRs has been extensively studied (Nichols et al., 2012).

### Recognition and Transportation of LPS via LBP-CD14-MD-2/TLR4

The combination of LPS and the MD-2/TLR4 complex triggers the host innate immune response, induces inflammation and cytokine production, and activates effector cells and complementary systems. In the TLR family, TLR4 is unique because it must form a dimer complex with MD-2 to capture its ligand LPS. Because multiple acyl chains of LPS lipid A are key to the MD-2/TLR4 complex, the LPS poly-acyl chains must be protected until they are incorporated into the MD-2/TLR4 complex. In this process, LPS-binding protein (LBP) and CD14, key auxiliary molecules, improve the efficiency of LPS transport, and sensitivity of detection (Ryu et al., 2017). LBP, a 60 kDa serum glycoprotein, is mainly produced by hepatocytes, lung, and gastrointestinal epithelial cells. Compared with those of other acute-phase proteins, the serum concentration of LBP increases slowly, peaking at ~2–3 days after acute infection. Surprisingly, a high concentration of LBP inhibits inflammation induced by LPS to some extent (Zweigner et al., 2001). Low concentrations of LBP have a high affinity for lipid A and thus promote formation of the LBP-LPS complex, which is subsequently transported to membrane CD14 (mCD14) or soluble CD14 (sCD14) (Ding and Jin, 2014). Soluble CD14 in the serum makes CD14-deficient cells, including most epithelial and endothelial cells, respond to LPS. Once CD14 binds to the LBP-LPS complex, LPS is transiently transferred to CD14, which has a hydrophobic pocket at its N-terminus that serves as the LPS binding site (Kelley et al., 2013). Transport of LPS to surface MD-2/TLR4 in the form of the LBP-LPS-CD14 complex is a prerequisite for TLR4 internalization and subsequent immune response (Tsukamoto et al., 2018). Notably, LBP expression in gingival epithelial cells (GECs) of the gingival-dental junction is significantly lower in CP patients than in healthy people (Ding and Jin, 2014). Research has shown that the addition of exogenous sCD14 substantially promotes the ability of human periodontal ligament stem cells (PDLSCs) to produce the inflammatory factors activated by *P. gingivalis*-LPS, including IL-6, IL-8 and chemokine (C-C motif) ligand 2 (CCL2) (Andrukhov et al., 2016). In fact, one study showed that both LBP and exogenous sCD14 could promote the internalization of TLR4, but the concentration of sCD14 required was higher than that of LBP (Tsukamoto et al., 2018).

In terms of the TLR-mediated innate immune response induced by Gram-negative bacteria, such as *P. gingivalis* and *C. burnetii* (the pathogen that causes Q fever), TLR2 and TLR4 are the most widely studied (Ramstead et al., 2016; Song et al., 2017). In particular, TLR2 requires heterodimerization with TLR1 or TLR6 to function properly, wherein the TLR2/TLR1 binding region is triacylated lipopeptides, such as Pam3CSK4, and the TLR2/TLR6 ligand is diacylated lipid/lipopeptides, such as lipoteichoic acid (LTA) (Nguyen et al., 2017). Based on this information, different subspecies of *C. burnetii* function differently in innate immune recognition; for example, *C. burnetii* Nine Mile is mediated by only the TLR1/TLR2 heterodimer, while *C. burnetii* 3262 is recognized by both TLR1/TLR2 and TLR2/TLR6 (Ammerdorffer et al., 2015). TLRs, as type I transmembrane proteins, are composed of a transmembrane structure, an extracellular amino terminus domain (recognizing PAMPs) and an intracellular Toll/interleukin-1 receptor (TIR) homology domain. To date, ten TLR subtypes have been found in humans, among which TLR2/TLR1, TLR2/TLR6, TLR4, TLR5, and TLR10 are cell surface receptors mainly identifying proteins or lipids, and TLR3, TLR8, and TLR9 bind the endoplasmic reticulum membrane, mainly identifying nucleic acids (Akira and Takeda, 2004). In conjunction with the downstream TLRs, some adaptor protein molecules recognize the TIR domain, including myeloid differentiation factor-88 (MyD88), MyD88-adapter-like/TIR-domain-containing adaptor protein (Mal/TIRAP), TIR-domain-containing adaptor-inducing IFN-β (TRIF), TRIF-related adaptor molecule (TRAM) and selective androgen receptor modulators (SARM). According to the adaptor protein recruited, TLR active pathways are divided into MyD88-dependent and TRIF-dependent pathways. TLR2/TLR1, TLR2/TLR6, TLR5, TLR7/TLR8, and TLR9 are all related to the MyD88-dependent pathway. TLR3 is activated through a TRIF-dependent pathway. TLR4 can activate the MyD88 pathway on the plasma membrane and then trigger the TRIF pathway by activating the TRAM adaptor on the endosomes (Liu et al., 2014; Chen et al., 2016). When MyD88 is activated as the downstream binding protein, nuclear factor kappa-light-chain-enhancer of activated B cells (NF-κB), activating protein (AP-1) and interferon response factors 1, 5, and 7 (IRF-1, IRF-5, IRF-7) became active, inducing the participation of inflammatory factor genes, while the TRIF pathway ultimately results in the sensitization of IRF-3 and activation of type I IFN-inducible genes (Roshan et al., 2016). The extracellular domain of TLR4 must initially bind to the MD-2 molecule to achieve the most powerful recognition and transmission of LPS. By forming a homodimer or a larger complex with the help of MD-2, TLR4 exhibits more binding sites for PAMPs on the cell membrane, significantly enhancing the activation of the NF-κB pathway (Visintin et al., 2001). The radioprotective 105 kDa protein (RP105, also termed CD180), originally discovered to protect B cells from radiation-induced apoptosis, is a specific inhibitor of TLR4. As the specific homolog of TLR4, CD180 is not the same Toll family receptor as other TLRs due to the loss of an intracellular signaling domain. Because it is physically associated with the MD-2-like molecule MD-1, substantial structural similarity but contrasting regulation exists between CD180 and TLR4 (Yang et al., 2018). Therefore, both MD-1/CD180 and MD-2/TLR4 are cell surface receptors that mediate LPS signaling pathways that have similar structures but opposite functions (Maeshima and Fernandez, 2013).

### Different Lipid a Structures of *P. gingivalis*-LPS Trigger Different Signal Pathways

Previous research reported that the mediation of TLR2 is essential for the loss of alveolar bone caused by *P. gingivalis* in animal models, and treatment of *E. coli*-LPS-tolerant bone marrow-derived macrophages (BMDM) with *P. gingivalis* resulted in upregulation of TLR2 expression and excessive tumor necrosis factor (TNF) production *in vitro* (Papadopoulos et al., 2013). In fact, questions regarding the leading signaling pathway activated in *P. gingivalis*-LPS-triggered immunoinflammatory reactions are controversial, as studies found that TLR4 exerts a dominant function, while others demonstrated that TLR2 is the primary contributor (Wang and Ohura, 2002). Some studies have reported that downstream cytokine changes are mainly caused by the NF-κB pathway, while others believe that the mitogen-activated protein kinase (MAPK) pathway and others, such as Jun N-terminal kinases (JNKs) and phosphatidylinositol 3-kinase (PI3K), are active during this process (Bainbridge and Darveau, 2001; Liu et al., 2013). For example, researchers found that *P. gingivalis*-LPS reduced the osteogenic polarization potential of PDLSCs via the TLR4-NF-κB signaling pathway. Moreover, blocking either TLR4 or NF-κB signaling hindered LPS-induced alveolar bone loss (Li et al., 2014). Other results indicated that *P. gingivalis*-LPS activates M1 and M2 macrophages mainly via TLR2, accompanied by the phenomena that high concentrations of LPS stimulate M1 macrophages to produce nitric oxide (NO), while low concentrations primarily increase the expression of TNF-α and IL-6 (Holden et al., 2014).

Regarding the involvement of the TLR4 pathway, penta-acylated *P. gingivalis*-LPS_1690_ is similar to classical hexa-acylated *E. coli*-LPS to some extent (Bozkurt et al., 2017). For example, both *P. gingivalis*-LPS_1690_ and *E. coli*-LPS elevate the expression of the LBP protein through TLR4 in human oral keratinocytes, among which the former induction is achieved via NF-κB and p38/MPAK pathways, whereas the latter regulation is controlled by the NF-κB, p38/MPAK and JNK signaling pathways (Ding et al., 2013). Another study showed that *P. gingivalis*-LPS distinctly enhanced IL-6 mRNA expression but downregulated the cell surface molecules TLR2 and TLR4, but not at the transcriptional level, while *E. coli*-LPS induced a similar but more obvious alteration than *P. gingivalis*-LPS (Andrukhov et al., 2014). However, some differences do exist between *P. gingivalis*-LPS_1690_ and *E. coli*-LPS based on cell types and their exact molecular biological activities. For example, *P. gingivalis*-LPS_1690_ significantly enhanced the transcription of NF-κB in mouse cementoblasts via TLR2, while *E. coli*-LPS was regulated by TLR4 (Nemoto et al., 2006). In addition, a study revealed that *P. gingivalis*-LPS_1690_ not only upregulated TLR4 expression in human gingival fibroblasts (hGFs) with positive correlations of dose and time, but also induced the expression of TLR2 (Herath et al., 2013a). Another study further demonstrated that the 16 kDa lipoprotein from *P. gingivalis*-LPS extracted by the phenol-water method activated hGFs to induce IL-8 production via the NF-κB pathway by means of TLR2, while *E. coli*-LPS was assisted by only TLR4 (Hashimoto et al., 2004).

**Figure 2:**
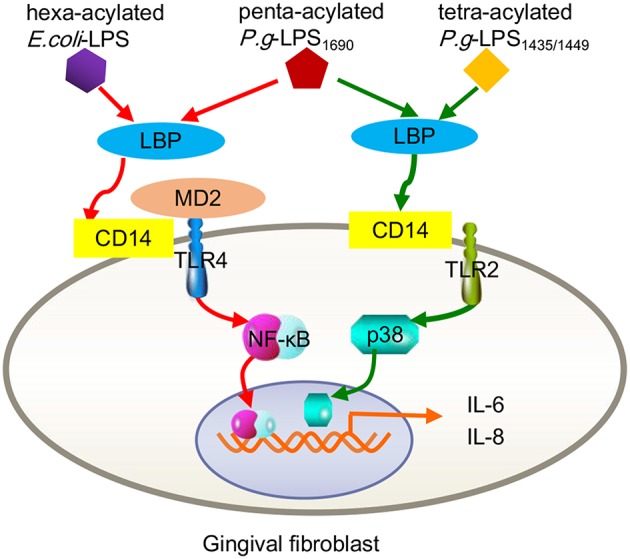
Contrast inflammatory signals in human gingival fibroblasts triggered by the different acylation structures of *P. gingivalis* LPS and *E. coli* LPS. *P.g*-LPS_1690_ upregulates the expression of IL-6 and IL-8, mainly via the MD-2/TLR4-NF-κB pathway. This process seemed to mimic hexa-acylated *E. coli*-LPS. Both *P.g* LPS_1435/1449_ and LPS_1690_ stimulate increased expression of TLR2, but *E. coli* LPS did not trigger TLR2. *P.g*-LPS_1435/1449_ primarily induced the p38/MAPK signaling pathway with little sensitization of the NF-κB pathway.

In fact, signaling pathways triggered by *P. gingivalis*-LPS are closely related to its acylated lipid A isomer and target cells. As mentioned earlier, *P. gingivalis* can be classified into two groups based on the different acylation configurations of lipid A, namely, penta-acylated LPS_1690_ and tetra-acylated LPS_1435/1449_, which differentially modulate innate host response and produce diverse substances, e.g., skin-antimicrobial peptide 1 (SAP1), inflammatory cytokines (IL-6, IL-8), and MMP-3 (Reife et al., 2006; Herath et al., 2011, 2013b). For example, *P. gingivalis*-LPS_1690_, but not LPS_1435/1449_, significantly increased TLR4, and MD-2 expression in hGFs at concentrations ≥0.1 μg/mL. Both of LPS_1690_ and LPS_1435/1449_ could significantly enhance the expression of TLR2 in hGFs, but the effect of LPS_1435/1449_ was stronger than that of LPS_1690_ (Herath et al., 2013a). Subsequent experiments demonstrated that *P. gingivalis*-LPS_1690_ significantly activated the NF-κB (mainly), p38/MAPK, and extracellular signal-regulated kinase (ERK)1/2 signaling pathways, while *P. gingivalis*-LPS_1435/1449_ primarily induced the p38/MAPK and ERK1/2 signaling pathways with little sensitization of the NF-κB pathway (Figure 2).

**Figure 3:**
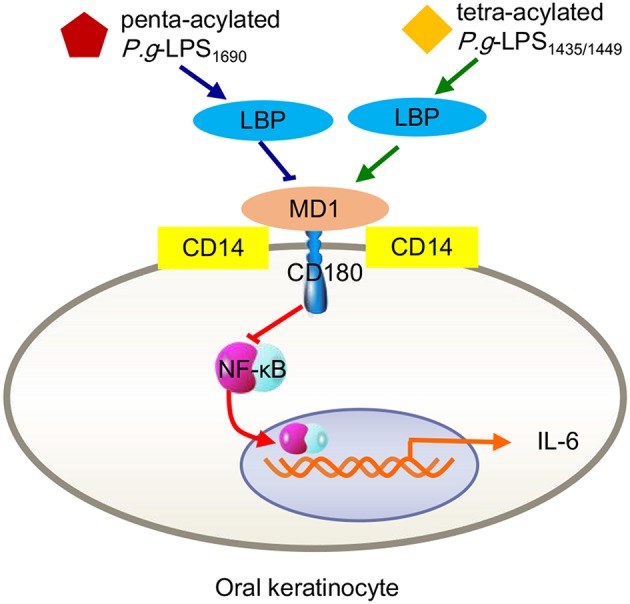
Inflammatory signals in human oral keratinocytes triggered by *P. gingivalis*-LPS heterogeneity. Either tetra-acylated *P.g*-LPS_1435/1449_ or penta-acylated LPS_1690_ downregulated the expression of IL-6 through the MD-2/TLR4-NF-κB pathway. Because MD-1/CD180 complexes negatively mediate the MD-2/TLR4 pathway, the degree of LPS_1435/1449_ downregulation via increasing MD-1 was more pronounced than that of LPS_1690_ via reducing MD-1.

Interestingly, *P. gingivalis*-LPS_1435/1449_ can block the expression of ELAM-1 induced by *E. coli*-LPS on human endothelial cells at the level of combination with the TLR4 receptor ectodomain, exhibiting a TLR4 antagonist ability (Coats et al., 2003). As a structure-determined function, researchers proposed that the shared MD-2 and MD-1 protein module mediates diverse biological functions through specific interactions with lipid structures (Nagai et al., 2005). As mentioned earlier, the MD-2/TLR4 compound-mediated immune response induced by bacterial LPS is closely associated with the combined MD-1/CD180 formation (Nagai et al., 2005). Due to the common features and functional interrelationship of MD-1/CD180 and TLR4/MD-2, the LPS-originating pathway can be restrained by MD-1/CD180 acting as an MD-2/TLR4 antagonist, which occurs in a wide ranges of cell types, including human embryonic kidney 293 (HEK293) cells, dendritic cells, and macrophages (Divanovic et al., 2005). In fact, this antagonistic activity results from the combination of the TLR4 SV1 splicing variant binding with MD-2, resulting in the absence of signaling molecules in the extracellular N-terminal domain (Coats et al., 2005). As another example, a recent study found that LBP alone can upregulate IL-6 expression in human oral keratinocytes, a process mediated by TLR2 and involving NF-κB, JNK/p38 and IRF. When *P. gingivalis*-LPS_1435/1449_ and LPS_1690_ interacted with LBP, both downregulated the expression of IL-6 in keratinocytes, and LPS_1435/1449_ was downregulated to a greater extent than LPS_1690_. Further studies verified that the expression levels of both CD180 and MD-1 were significantly increased after LPS_1435/1449_ bound LBP, while the combination of LPS_1690_ and LBP upregulated CD180 expression but decreased MD-1 expression. This result provided a precise explanation for the previously mentioned result that the downregulation of IL-6 caused by LPS_1435/1449_ binding with LBP was more obvious than that of LPS_1690_ (Figure 3). The above results lead to the conclusion that MD-1/CD180 acts as a fine-tuning mediator of the regulation of *P. gingivalis*-LPS heterogeneity induced signaling pathways (Ding et al., 2017).

In summary, we can draw the following conclusions: regarding the TLR2 pathway, either *P. gingivalis* LPS_1690_ or LPS_1435/1449_ serves as an excitomotor; in particular, LPS_1690_ is a strong TLR4 agonist, while LPS_1435/1449_ is only a weak stimulant, and more precisely, a potent antagonist of TLR4 (Herath et al., 2013a). *In vitro* experiments indicated that the response to *P. gingivalis*-LPS varied considerably depending on the cell types examined or the type of LPS produced (especially in some experiments, as LPS extracted from *P. gingivalis* was not completely pure; Darveau et al., 2004). Furthermore, both *P. gingivalis* LPS_1690_ and LPS_1435/1449_ increased the amount of IFN-γ in whole blood cell cultures (WBCCs) from CP patients, and the former showed higher secretion results. Moreover, *P. gingivalis*-LPS_1690_ could trigger the abnormal whole blood cell secretion of IL-10 in healthy individuals, while *P. gingivalis*-LPS_1435/1449_ could not, further proving that different types of *P. gingivalis*-LPS induce unequivocal changes in the blood systems of CP patients (Nogueira-Filho et al., 2014). On the other hand, considering the complex circumstances of local and systemic hosts *in vivo*, one study demonstrated that both LPS_1690_ and LPS_1435/1449_ were effective stimulators of inflammation, and LPS_1435/1449_ was more effective than LPS_1690_ but less harmful than *E. coli*-LPS at the site of inoculation (Liu et al., 2008). Currently, how host cells protect against different *P. gingivalis* phenotypes dependent on the microenvironmental condition is not fully understood (Ding et al., 2017). However, changing the heterogeneous lipid A structure to adapt to tissue-specific cellular receptor signaling pathways has clearly been demonstrated to be a mechanism by which *P. gingivalis* avoids the host's innate immune attack and accesses the periodontal tissue (Olsen and Singhrao, 2018).

## Effect of Gingipains on *P. gingivalis* Virulence

### Classification of Gingipains

**Figure 4:**
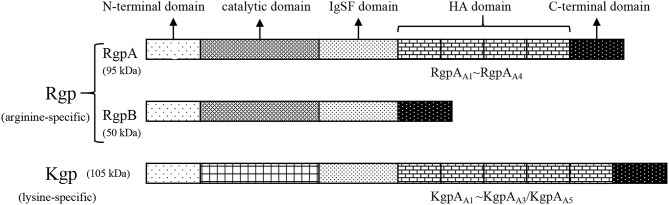
Structure of gingipains. Gingipains are divided into Rgp (arginine-dependent gingipain R) and Kgp (lysine-dependent gingipain K). Rgp has been further subdivided into RgpA and RgpB. The molecular weight of RgpA, RgpB and Kgp is 95, 50, and 105 kDa, respectively. Gingipains are primarily made up of the following components: the signal peptide, the N-terminal domain, the catalytic domain (CD), the immunoglobulin superfamily-like domain (IgSF), the hemagglutinin/adhesion (HA) domain, and the C-terminal domain. The structure of RgpB is the simplest without HA domains. RgpA has four HA domains (called RgpA_A1_ to RgpA_A4_) located in the middle of the IgSF and C-terminal domain. Kgp also has 3–5 such domains (called KgpA_A1_ to KgpA_A5_) in the light of different bacterial strains.

Gingipains (named after *P. gingivalis* clostripain), belonging to the cysteine protease family and existing in the outer membranes, vesicles, and extracellular structures of *P. gingivalis*, are important virulence factors that exert the vital function of mediating the interaction between *P. gingivalis* bacteria and hosts (Yongqing et al., 2011). Gingipains can be divided into two categories: arginine-dependent gingipain R (Rgp) and lysine-dependent gingipain K (Kgp). Together, the two kinds of *P. gingivalis* gingipains complete 85% of the proteolysis outside the cell (de Diego et al., 2014). Rgp has been further subdivided into RgpA and RgpB on the basis of structure. The molecular weights of RgpA, RgpB, and Kgp, which are coded by the *rgpA, rgpB*, and *kgp* genes, are 95, 50, and 105 kDa, respectively. Collectively, these enzymes are primarily composed of the following components: a signal peptide, an N-terminal domain, a catalytic domain (CD), an immunoglobulin superfamily-like domain (IgSF), a hemagglutinin/adhesion (HA) domain and a C-terminal domain (Nakayama, 2015). Among these proteins, the structure of RgpB is the simplest, as it has no HA domain, while RgpA contains four HA domains (called RgpA_A1_-RgpA_A4_) located in the middle of the IgSF and C-terminal domains. Kgp also has 3 to 5 such domains (called KgpA_A1_-KgpA_A5_) in different bacterial strains (de Diego et al., 2013; structure of gingipains shown in Figure 4). The CDs of RgpB and RgpA are highly homologous in terms of their amino acid sequences, but no proteins with CD structures similar to those of Kgp have been reported. Based on analysis of the high-resolution crystal structure of Kgp competent fragments, the key catalytic mechanism of Kgp probably lies in the requirement of a triplet (C^477^-H^444^-D^388^) instead of a cysteine-histidine dimer (de Diego et al., 2014; Gorman et al., 2015). In addition, dimerization of the pro-domain also plays a substantial role in the specific latency mechanism of Kgp (Pomowski et al., 2017). In an animal experiment designed to test the efficacy of three gene vaccines (*rgpA, rgpB*, and *kgp*) in treating peri-implant inflammation, the *kgp* and *rgpA* DNA vaccines enhanced immune responses and significantly retarded alveolar bone loss *in vivo*, whereas the *rgpB* vaccine was ineffective (Guo et al., 2014). Given the different biological properties of RgpA, RgpB, and Kgp, the virulence rankings of the three gingipains explored using periodontitis model mice was Kgp > or = RgpB >> RgpA (Pathirana et al., 2007).

### Secretion and Activation of Gingipains

The biosynthesis of gingipains (secretion and activation) is a complicated process that has not yet been fully explored. The overall process is as follows: when gingipains have just been translated, they are present in the form of an inactive zymogen to block the unwanted proteolytic activity inside the cell. After posttranslational processing, gingipains are converted into catalytically active proteases and then transported to the extracellular environment. Although the process of converting from an inactive zymogen to a catalytic gingipain is not fully defined, acetylated-lysine residues were found in the structures of these three mature proteases, suggesting that acetylation is a potential mechanism underlying gingipain activation and maturation (Ren et al., 2017). Herein, Vim A must be mentioned as a necessary acetyltransferase in the maturation process of gingipains to modulate the virulence of *P. gingivalis*, and it is indispensable for the maturation of RgpA (Aruni et al., 2013). However, RgpB and Kgp can be synthesized and secreted via a Vim A-independent pathway, which accounts for the late-onset protease activity observed in Vim A-deficient mutants (Dou et al., 2015). A recent study showed that PG1842 can act as an acetyltransferase to replace Vim A in the process of RgpB acetylation. In any case, acetylation is definitively a requisite posttranslational protein modification during the process of gingipain maturation (Mishra et al., 2018).

### Contribution of Gingipains to Pathogenic Polymicrobial Biofilms

Regarding the status of *P. gingivalis* during oral biofilm, gingipain protease activity affects the composition of multimicrobial biofilm quantitatively and qualitatively (Hocevar et al., 2018). Gingipains also function as ligands in the coaggregation of *P. gingivalis* with other oral bacteria, such as *T. denticola*, to promote the colonization of *P. gingivalis* in dental plaque (Ito et al., 2010). In a biofilm model consisting of ten subgingival bacteria, the most prominent outcome of replacing the normal strain with a *P. gingivalis* Rgp/Kgp mutant was the change in the *T. denticola* three-dimensional distribution throughout the biofilm. Among these proteins, Rgp enhanced the growth of *T. denticola*, and Kgp promoted the accumulation of *T. denticola* in the biofilm. This synergistic effect was shown to be beneficial to not only the survival and virulence of biofilm colonies but also to their ability to form the red bacterial complex (Bao et al., 2014). Kgp has been proven to play a very important role in the shedding and reduction of biofilms, improving the competitive advantage of *P. gingivalis* in plaque biofilms. In addition, both RgpA and Kgp participate in *P. gingivalis* adhesion to oral epithelial cells, aggregating with other bacterial species (Sakanaka et al., 2016).

### Proteolytic Action of Gingipains in the Host TLR-Mediated Immune Response

By virtue of the proteolytic action of gingipains, *P. gingivalis* cleaves or degrades a variety of host proteins to escape immune defense, including immunomodulatory proteins, signaling pathway regulatory proteins, and adhesion molecules (Barth et al., 2013; Hocevar et al., 2018). *P. gingivalis* can also recognize NLRs in a gingipain-independent manner, activate NLR pyrin domain-containing 3 (NLRP3)-modified inflammatory corpuscle and release the inflammatory factors IL-1β and TNF-α, inducing an inflammatory response in the host. Interestingly, gingipains released by *P. gingivalis* itself can degrade mature IL-1β and TNF-α, weakening the inflammatory response of the host to some extent. Both the positive and negative immunomodulatory effects of *P. gingivalis* on the host mentioned above are actually beneficial to long-lasting colonization in periodontal tissues (Jung et al., 2015). For instance, gingipains inhibit the cellular PI3K/Akt signaling pathway by cleaving extracellular PI3Kp85α-associated membrane proteins, thereby achieving immune regulation of GECs, and this process is independent of virulence factor invasion (Nakayama et al., 2015). The purified gingipains RgpA and Kgp downregulate mCD14 expression in a time- and concentration-dependent manner, resulting in low responsiveness of macrophages to *P. gingivalis* infection (Wilensky et al., 2015). This reduced mCD14 expression relies on the presence of the HA domain, causing RgpA and Kgp to exhibit the shedding enzyme effect, while RgpB is not active because of its lack of the HA domain. The weakening of CD14 efficacy is not only beneficial for the reproduction of eosinophilic bacteria in bacterial biofilms but also leads to more severe chronic inflammation (Olsen et al., 2017).

**Figure 5:**
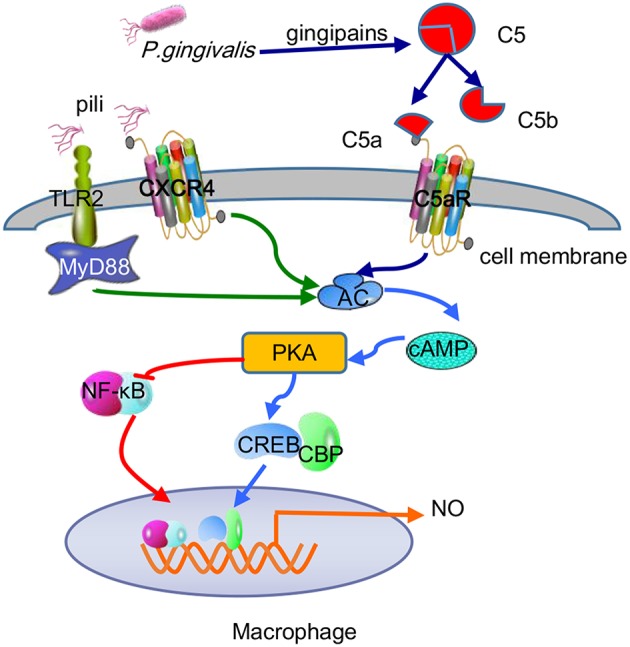
Antibactericidal mechanisms of C5a-TLR2-CXCR4 cross-talk induced by *P. gingivalis* in macrophages. *P. gingivalis* pili activated the TLR2-MyD88-dependent pathway and caused a small amount of cAMP production, while C5a-C5aR activation mediated by *P. gingivalis* gingipain-degradation of C5 synergistically enhances the production of cAMP. The combination of pili and CXCR4 helped maximize cAMP production via C5a-TLR2 cross-talk. The continuous increase in cAMP activated PKA to mediate CREB capturing the limited binding sites of CBP and inhibited NF-κB, thus reducing macrophage-forming NO and destroying the bactericidal function.

For the most part, *P. gingivalis* withstands the bactericidal lytic activity in blood serum because gingipains are the main force in combating complement systems, which can be applicated in hosts via three mechanisms: classical, lectin, and alternative. The initial stages of these three pathways differ, but the final outcomes involve the insertion of membrane attack complexes into cell membranes, inducing the generation of chemicals and phagocytosis of targeted Gram-negative bacteria (Ricklin et al., 2010). *P. gingivalis*-specific gingipains decompose C5 into C5a and C5b in two ways, by directly exerting C5 convertase-like activity and activating thrombin to replace C5 convertase by activating prothrombin (Hajishengallis et al., 2013a). It is worth noting that gingipains induce the polarization of M1 macrophages as a regulatory factor, thereby facilitating *P. gingivalis* infection via a C5a-mediated pathway (Hou et al., 2017). C5a is an effective chemotactic agent and phagocyte activator that is unfavorable for *P. gingivalis*. However, *P. gingivalis* itself has some resistance to complement dissolution due to the anionic polysaccharide structure of lipid A anchored on the surface (Olsen et al., 2017). In addition, RgpA hijacks and adsorbs C4b-binding protein, a complement physiological regulator, on the surface of *P. gingivalis*, inhibiting the classical and lectin pathways of the complement system (Hertz et al., 2018). More importantly, recent studies have found an important mechanism of combined action between the C5a receptor (C5aR) and TLR2 in macrophages to promote the adaptability of *P. gingivalis* (Wang et al., 2010). While the pili or lipoproteins of *P. gingivalis* activate TLR2/TLR1 on macrophages and induce a small amount of cyclic adenosine monophosphate (cAMP), C5a-C5aR synergistically enhances the weak cAMP response activated by TLR2/TLR1 alone by stimulating calcium-dependent intracellular signaling, resulting in a large amount of cAMP. Moreover, the continuous increase in cAMP activates cAMP-dependent protein kinase A (PKA) in macrophages and destroys the bactericidal function of inducible NO synthase (iNOS), which is dependent on NF-κB (Wang et al., 2010). In fact, the cross-talk mechanism of C5aR-TLR2 resulting in maximal macrophage cAMP production stimulated by *P. gingivalis* is specifically attributable to the assistance of CXC-chemokine receptor 4 (CXCR4), although the coassociation of C5aR and TLR2 in lipid rafts can effectively increase cAMP production in a CXCR4-independent manner (Wang et al., 2010). The regulatory action of the cAMP/PKA pathway on cell activity depends on mainly the binding of phosphorylated cAMP response element binding protein (CREB) to nuclear coactivator CREB-binding protein (CBP) (Dyson and Wright, 2016). Because intracellular CBP is limited, CREB (Ser133) phosphorylated by PKA and NF-κB p65 (Ser276) competently binds to CBP. Because cAMP/PKA mediates CREB to capture the binding CBP sites, the NF-κB pathway is suppressed (Figure 5). Moreover, upon the addition of glycogen synthase kinase 3β (GSK3β) inhibitors, the iNOS and NO produced by the coactivation of *P. gingivalis*-excited C5a/TLR2 were reduced to some extent but not completely inhibited, demonstrating that GSK3β partially promotes the iNOS signaling pathway. On the other hand, the aggregation of PKA also inhibits IRF-1, which is the key to the IFN-γ-mediated synergistic promotion of iNOS transcription (Salim et al., 2016). Therefore, the cross-talk between C5aR and TLR2 induces a cAMP-dependent immune overthrow, which leads to the reduction of iNOS and weakening of the bactericidal efficacy of macrophages. At the same time, GSK3β (Ser9) is inactivated by PKA phosphorylation, which is key to regulating proinflammatory and anti-inflammatory factors, inhibiting the production of TLR-mediated proinflammatory mediators (e.g., IL-6, TNF-α, IL-12, and IFN-γ) in host cells (Martin et al., 2005). The C5a-C5aR signaling axis also inhibits TLR4-induced IL-12p35, IL-12/23p40, and IL-23p19 expression at the transcriptional level in macrophages and downregulates IL-12p70 and IL-23 at the translation level by the PI3K and ERK1/2 signaling pathways, thereby alleviating latent tissue damage regulated by effector Th1 and Th17 cells (Weaver et al., 2007).

### Transpeptidation Function of Gingipains

In addition to the powerful protein degradation function mentioned above, gingipains have recently been found to have an undeniable transpeptidation effect, which results from the transfer of several amino acids between peptide chains. Considering that heme must be ingested to provide iron and protoporphyrin IX and thus ensure viability and virulence (Ohya et al., 2016), *P. gingivalis*, an obligate anaerobic bacterium located in subgingival plaque, utilizes the transpeptidase activity of gingipains to extract the required nutrients from human hemoglobin (Smalley and Olczak, 2017). During this process, small peptides such as glycylglycine (GlyGly) not only enhance the proteolytic activity of gingipains but also act as receptor molecules participating in gingipain-catalyzed transpeptidation. In general, the trans-peptide reaction is much more efficient than the corresponding hydrolysis reaction, causing simple pathogenic proteins to become “transpeptidases” in a short time, which may induce the destruction of immune tolerance and trigger autoimmune diseases (Zhang et al., 2018). In addition, Kgp may not only act as a sensor of the hemoglobin concentration in the environment, thereby regulating the acetylation mode of lipid A in *P. gingivalis*-LPS, but also directly participate in the aggregation and degradation of hemoglobin, affecting its type, growth, and infection of *P. gingivalis* (Smalley and Olczak, 2017).

## Effect of Fimbriae/Pili on *P. gingivalis* Virulence

### Structure and Genotype of Pili

Pili are filamentous structures located on the *P. gingivalis* surface that enhance the bacterial adhesion to multiple types of surfaces, such as the extracellular matrix, host cells and other bacteria, and take part in the formation of biofilm (Nagano et al., 2013). *P. gingivalis* pili can be exhibited in at least two forms, namely, the major FimA and the minor Mfa, both of which regulate bacterial dependence on various molecules and oral substrates and are important for biofilm formation (Nagano et al., 2012). The major proteins, which protrude ~3 μm above the surface, are composed of the FimA protein subunit (the main *P. gingivalis* pilus subunit) and are encoded by the *fimA* gene, while the minor subunits, which are also called short fimbriae, are composed of mainly Mfa1 structural subunit proteins and are encoded by the *mfa1* gene, and their lengths differ from 60 to 500 nm (Nakano and Amano, 2013). In addition to the primary Mfa1 protein, mature pili also have affiliated Mfa2-5 proteins. Mfa2 plays an anchor role, while Mfa3 can bind with Mfa1/2/4/5 *in vitro*, connecting with other pilus subunits as a binding protein (Ikai et al., 2015). Recent data indicate that the C-terminal domain of Mfa1, rather than Mfa3, affects the aggregation and maturation of downstream pilus proteins (Hall et al., 2018); however, the aggregation of Mfa1 does not depend on other pilus proteins but requires the proteolytic action produced by the gingipains RgpA and RgpB (Lee et al., 2018). Based on nucleotide sequence differences between the translational reading frame on the gene *fimA, P. gingivalis* is divided into six genotypes (type I to V and Ib). Among these genotypes, the *fimA* genotypes II and IV are widely distributed in periodontitis isolates (Nagano et al., 2018). The adhesive and invasive abilities of epithelial cells in patients with type II *fimA* clones were shown to be significantly enhanced compared with those of other *fimA* clones, suggesting that these clones are most closely associated with CP (Enersen et al., 2008). A recent study showed that type II *fimA* genotype is also detected at high rates in patients with both periodontitis and rheumatoid arthritis (RA) (Ayala-Herrera et al., 2018).

### Role of Fimbriae/Pili in TLR-Mediated Host Immune Response

Adhesion is an indispensable process in the pathological development of periodontitis. Because the pili are the most prominent structure on the surface of bacteria, it is highly likely that their attachment is the first step in the reaction between microorganisms and the host (Mantri et al., 2015). It has been confirmed that pili can mediate the attachment of *P. gingivalis* to hydroxyapatite, hGFs and epithelia (Sojar et al., 2002; Baek et al., 2013). *Porphyromonas gingivalis* long fimbrial proteins were capable of activating human GECs through TLR2 with a complex of sCD14 and LBP and significantly upregulating IL-8 expression and NF-κB activation, which were involved in bone resorption (Kusumoto et al., 2004). *Porphyromonas gingivalis* minor pili not only enhanced the bone resorption of osteoclasts by producing IL-1β, TNF-α, and IL-6 but also promoted the differentiation of osteoclast precursor cells. The addition of anti-TLR2 antibody significantly inhibited the formation of osteoclasts caused by short fimbriae, while anti-TLR4 antibody did not obviously block pit formation on dentine blocks (Hiramine et al., 2003). Moreover, similar results demonstrated that the 67-kDa minor pili on the *P. gingivalis* surface stimulated the expression of TNF-α, IL-1α, IL-1β, and IL-6 cytokines in macrophages via the TLR2/complement receptor 3 (CR3) pathway, which showed the vital function of regulating immunity and mediating tissue damage during the development of periodontitis (Wang et al., 2007). Similar to CD14 regulating the sensitivity of TLR4-MD2 combination with LPS, *P. gingivalis* fimbriae upregulated IL-6 expression via the TLR2-p38/MAPK pathway in human monocytes, while LBP improved the reaction (Pollreisz et al., 2010). In addition, although sCD14 is a necessary molecule to recognize *P. gingivalis* by epithelial cells, mCD14 can cause a more violent reaction in response to activation of downstream pathways, producing IL-6, IL-8, granulocyte-macrophage colony-stimulating factor (GM-CSF), and TNF-α (Eskan et al., 2007).

It was shown that the different combinations between the coreceptor and TLR2 directly affect the activation of microbial molecules and TLR2; for instance, CXCR4 acts as a pattern recognition receptor and is also a coreceptor for TLR2 (van Bergenhenegouwen et al., 2013). The combination of pili and CXCR4 blocks the production of TNF-α mediated by NF-κB activation and simultaneously promotes IL-10 production. Further studies indicated that cross-talk exists between the TLR2/CXCR4 signaling pathway and the inhibition of the NF-κB pathway following TLR2 recognition of the pili. The key factor of this mechanism is that cAMP-dependent PKA inhibits the MyD88-dependent antibacterial pathway induced by TLR2 activation, which is the signaling transducer downstream of CXCR4 as mentioned before, reducing macrophage-forming NO and enhancing *P. gingivalis* against host clearance (Hajishengallis et al., 2008). In addition, the recognition of CXCR4 by *P. gingivalis* pili can activate the β2 integrin CR3 induced by PI3K in a TLR2-dependent manner, representing another cellular adhesion signaling pathway widely distributed in neutrophils, natural killer (NK) cells and macrophages. Upon CR3 activation, the safe entry of *P. gingivalis* into macrophages is mediated, simultaneously inhibiting the production of IL-12p70 and enhancing its capacity to evade death (Hajishengallis et al., 2013b).

**Figure 6:**
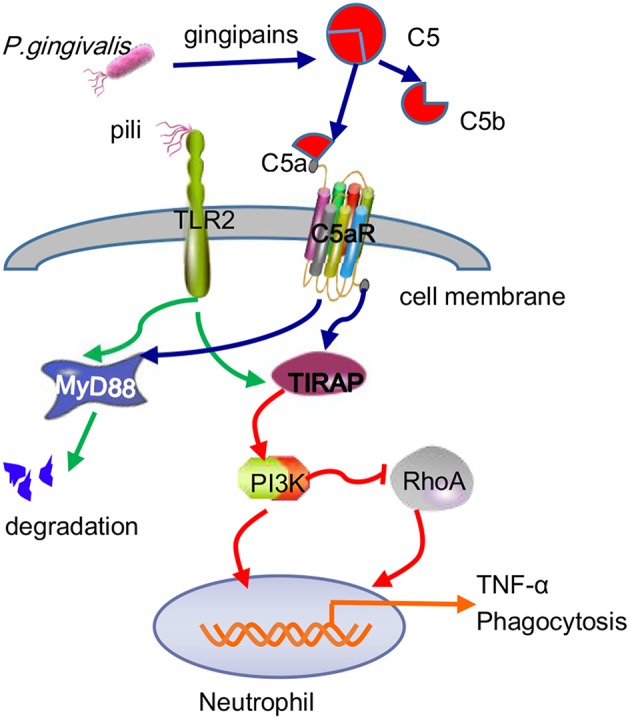
Antibactericidal mechanisms of C5a-TLR2 cross-talk induced by *P. gingivalis* in neutrophils. The C5aR-TLR2 cross-talk activated by *P. gingivalis* pili induced degradation of MyD88 in neutrophils. Without MyD88, the coassociation of C5aR-TLR2 promoted *P. gingivalis* infection through activation of the TIRAP-dependent PI3K signaling pathway, not only producing the inflammatory cytokine TNF-α but also blocking RhoA activation and actin polymerization to inhibit the maturation of phagosomes, thus blocking *P. gingivalis* phagocytosis.

Many studies have concluded that TLR2-MyD88 is a classical inflammatory pathway that mediates the periodontal tissue destruction caused by *P. gingivalis* (Burns et al., 2010). Recently, the TLR2-MAL/TIRAP-PI3K pathway was shown to promote *P. gingivalis* infection-driven alveolar bone resorption *in vivo*, even in the absence of the typical TLR2 adapter protein MyD88. At the same time, *in vitro* experiments demonstrated that activation of the TLR2-PI3K signal caused neutrophils to produce the proinflammatory cytokine TNF-α, thus inhibiting the maturation of phagosomes and prolonging survival time in host cells (Makkawi et al., 2017). While MyD88 contributes to the defense of neutrophils against *P. gingivalis*, pathogens can eliminate this host anti-infection mechanism by inducing the degradation of MyD88 in both humans and mice. The ability of *P. gingivalis* to degenerate MyD88 in neutrophils has been proven to be achieved through the C5aR-TLR2 cross-talk mechanism, in which the ubiquitin-proteasome and smad ubiquitin regulatory factor 1 (Smurfl) are involved, rather than via cAMP-dependent PKA (Maekawa et al., 2014). Furthermore, the MyD88-independent signaling pathway was also shown to work together with C5aR-TLR2 to promote *P. gingivalis* infection, during which PI3K acts as an effector of C5aR-TLR2 induced by the adaptor TIRAP (Hajishengallis, 2015) and blocks Ras homolog gene family member A (RhoA) activation and actin polymerization, thereby inhibiting *P. gingivalis* phagocytosis (Figure 6). In this regard, this result differs from the C5aR-TLR2 cross-talk in macrophages mentioned earlier.

## Conclusion

The oral cavity is an open microecological environment with more than 700 species of microorganisms that normally maintain a dynamic balance with the host's immune system. When the balance between bacteria and the host is perturbed, opportunistic pathogens, including periodontal pathogens, will become dominant. Periodontitis caused by periodontal pathogens destroys the epithelial junction between the teeth and periodontal tissue, forming a periodontal pocket. Furthermore, the periodontal pocket provides an anaerobic and nutrient-rich growth microenvironment for periodontal pathogens to survive and spreading (Wolf and Lamster, 2011). As the most important pathogen of periodontal tissue infection, *P. gingivalis* can directly destroy periodontal tissues by secreting toxic factors such as LPS, gingipains and pili, and these important virulence factors can activate a wide range of host immune cells in periodontal tissues, triggering a local immune response, allowing the defense cells to release numerous inflammatory mediators and leading to secondary damage to the periodontal tissue. Regarding periodontitis pathogenesis, TLRs, as PAMP recognition receptors, can mediate the inherent immunological reactions of the host to *P. gingivalis*, which is the basis of adaptive immunity and plays a vital role during the occurrence and development of periodontitis (Nakayama and Ohara, 2017). This review therefore details how the most critical virulence mediators of *P. gingivalis* trigger host defense cells and regulate the microbial-host immune-inflammatory responses by interacting with TLRs at the molecular biology level, hopefully providing an opportunity to more clearly understand the pathogenesis of periodontitis.

Considering the different experimental methods, cell types, reagents, and other experimental details used by different scholars, contradictions and controversies in the experimental results remain. To date, we still do not fully understand how *P. gingivalis* can not only induce bodily inflammation but also escape the host immune surveillance and flourish under the microecologically imbalanced system by fully exploiting its key virulence molecules. In addition, other toxicity factors produced by *P. gingivalis* also play substantial roles in the pathogenesis of periodontitis, such as capsules activating the host complement system (Singh et al., 2011), the hemophore HmuY helping *P. gingivalis* capture and internalize heme from the host (Smalley et al., 2011), and outer membrane proteins adhering to the host outer membrane (Chen et al., 2011). Regarding other virulence factors, their specific pathogenic mechanisms and how they regulate host immunity through TLRs will be further summarized. Elucidating the unique pathogenic mechanism of *P. gingivalis* virulence factors is an arduous task, and a substantial amount of work remains and requires the joint efforts of scholars. We believe that with the rapid development of biomedicine, these problems will eventually be solved to provide more effective clinical therapy for patients with periodontitis and periodontal-related systemic diseases.

## Author Contributions

LJ analyzed literature and wrote the paper. NH, JD, LG, ZL, and YL edited and approved the manuscript.

### Conflict of Interest Statement

The authors declare that the research was conducted in the absence of any commercial or financial relationships that could be construed as a potential conflict of interest.
